# IDENTIFYING BRIDGE SYMPTOMS AMONG DEPRESSIVE SYMPTOMS IN CHINESE OLDER ADULTS: THE ROLE OF EATING CULTURE

**DOI:** 10.1093/geroni/igad104.3403

**Published:** 2023-12-21

**Authors:** Zhongfang Yang, Junwen Yu, Tiantian Hu, Zheng Zhu

**Affiliations:** Fudan University, Shanghai, Shanghai, China (People’s Republic); Fudan University, Shanghai, Shanghai, China (People’s Republic); Fudan University, Shanghai, Shanghai, China (People’s Republic); New York University, New York City, New York, United States

## Abstract

**Introduction:**

Identifying bridge symptoms among Chinese older adults with depressive symptoms is crucial for enhancing the precision and individualization of interventions.

**Aim:**

The aim of this study was to identify the bridge symptoms and to explore the intercorrelation between depressive symptoms clusters in Chinese older adults.

**Methods:**

Depressive symptoms were measured in 2014, 2016, and 2018 using the nine-item version of Center for Epidemiological Studies Depression Scale, and analyzed with cross-lagged panel dynamic network and centrality examination.

**Results:**

Total of 3,438 participants were included. “D5: Had poor appetite” and “D9: Enjoy life” reported the highest value of outstrength (rnegative=0.063; rpositive=0.048) and the highest value of bridge strength (rnegative=0.029; rpositive=0.024) in negative and positive affects cluster. The strongest path is from D5 to D9 (β=-0.02).

**Discussion:**

To explore the potential mechanism of depressive symptoms in Chinese older adults, identifying the bridging symptoms between the positive affect clusters and negative symptoms clusters of depressive symptoms may be needed.

**Implications for Practice:**

More insights into the relationship between eating culture and the potential mechanisms could be provided to aid in the development of culturally sensitive interventions for Chinese older adults with depressive symptoms.

